# β_2_-Adrenergic Receptor (*ADRB2*) Gene Polymorphisms and Risk of COPD Exacerbations: The Rotterdam Study

**DOI:** 10.3390/jcm8111835

**Published:** 2019-11-01

**Authors:** Leila Karimi, Lies Lahousse, Mohsen Ghanbari, Natalie Terzikhan, André G. Uitterlinden, Johan van der Lei, Guy G. Brusselle, Bruno H. Stricker, Katia M. C. Verhamme

**Affiliations:** 1 Department of Medical Informatics, Erasmus University Medical Center, Doctor Molewaterplein 40, 3015 GD Rotterdam, The Netherlands; l.karimi@erasmusmc.nl (L.K.); j.vanderlei@erasmusmc.nl (J.v.d.L.); k.verhamme@erasmusmc.nl (K.M.C.V.); 2 Department of Bioanalysis, Ghent University, Ottergemsesteenweg 460, 9000 Ghent, Belgium; lies.lahousse@ugent.be; 3 Department of Epidemiology, Erasmus University Medical Center, Doctor Molewaterplein 40, 3015 GD Rotterdam, The Netherlands; m.ghanbari@erasmusmc.nl (M.G.); n.terzikhan@erasmusmc.nl (N.T.); Guy.Brusselle@uzgent.be (G.G.B.); 4 Department of Genetics, School of Medicine, Mashhad University of Medical Science, 9177899191 Mashhad, Iran; 5 Department of Respiratory Medicine, Ghent University Hospital, Corneel Heymanslaan 10, 9000 Ghent, Belgium; 6 Department of Internal Medicine, Erasmus University Medical Center, Doctor Molewaterplein 40, 3015 GD Rotterdam, The Netherlands; a.g.uitterlinden@erasmusmc.nl; 7 Department of Respiratory Medicine, Erasmus University Medical Center, Doctor Molewaterplein 40, 3015 GD Rotterdam, The Netherlands

**Keywords:** chronic obstructive pulmonary disease, inhaled β_2_-agonists, exacerbations, *ADRB2 gene*

## Abstract

The role of the β_2_-adrenergic receptor (*ADRB2*) gene in patients with chronic obstructive pulmonary disease (COPD) is unclear. We investigated the association between *ADRB2* variants and the risk of exacerbations in COPD patients treated with inhaled β_2_-agonists. Within the Rotterdam Study, a population-based cohort study, we followed 1053 COPD patients until the first COPD exacerbation or end of follow-up and extracted rs1042713 (16Arg > Gly) and rs1042714 (27Gln > Glu) in *ADRB2*. Exposure to inhaled β_2_-agonists was categorized into current, past, or non-use on the index date (date of COPD exacerbation for cases and on the same day of follow-up for controls). COPD exacerbations were defined as acute episodes of worsening symptoms requiring systemic corticosteroids and/or antibiotics (moderate exacerbations), or hospitalization (severe exacerbations). The associations between *ADRB2* variants and COPD exacerbations were assessed using Cox proportional hazards models, adjusting for age, sex, use of inhaled corticosteroids, daily dose of β_2_-agonists, and smoking. In current users of β_2_-agonists, the risk of COPD exacerbation decreased by 30% (hazard ratio (HR); 0.70, 95% CI: 0.59–0.84) for each copy of the Arg allele of rs1042713 and by 20% (HR; 0.80, 95% CI: 0.69–0.94) for each copy of the Gln allele of rs1042714. Furthermore, current users carrying the Arg16/Gln27 haplotype had a significantly lower risk (HR; 0.70, 95% CI: 0.59–0.85) of COPD exacerbation compared to the Gly16/Glu27 haplotype. In conclusion, we observed that the Arg16/Gln27 haplotype in *ADRB2* was associated with a reduced risk of COPD exacerbation in current users of inhaled β_2_-agonists.

## 1. Introduction

Chronic Obstructive Pulmonary Disease (COPD) is a common disease, which is characterized by a persistent expiratory airflow limitation that is usually progressive [1]. Exacerbations of respiratory symptoms frequently occur in COPD patients and are triggered by environmental pollutants, respiratory infections with bacteria or viruses, and unknown factors [1]. Inhaled β_2_-receptor agonists are one of the main classes of bronchodilators used to treat airflow obstruction [1]. The β_2_-adrenergic receptor is a member of the G protein-coupled transmembrane receptors widely located on airway smooth muscle cells that mediate relaxation and thus bronchodilation [2,3], and therefore is an important drug target in COPD treatment. The gene encoding the β_2_-adrenergic receptor, *ADRB2*, is a small intron-less gene on chromosome 5q31-32 [2]. Multiple single nucleotide polymorphisms (SNPs) in this gene have been described [2]. Two of these SNPs code for amino acid changes at positions 16 [arginine to glycine (16Arg > Gly); rs1042713] and 27 [glutamine to glutamic acid (27Gln > Glu); rs1042714], both of which are common variants and have previously been studied [4,5].

There is inconsistent evidence from previous studies on the association between *ADRB2* polymorphisms and treatment response to inhaled β_2_-agonists on COPD exacerbations [6,7,8], short-term bronchodilator response (BDRs) [9,10], and long-term changes in forced expiratory volume in 1 s (FEV1) in patients with COPD [10]. In addition, most studies assessed the effect of each SNP in isolation but not the combined effect of their haplotypes.

In this study, our main objective was to investigate whether two functional SNPs of the *ADRB2* gene, rs1042713 (16Arg > Gly) and rs1042714 (27Gln > Glu), and their haplotypes were associated with risk of exacerbations in COPD patients treated with inhaled β_2_-agonists.

## 2. Methods

### 2.1. Setting and Study Population

The current study was conducted using data from the Rotterdam Study, an ongoing prospective population-based cohort study among inhabitants of the Ommoord district of Rotterdam, the Netherlands. The rationale and design of the Rotterdam Study have been described elsewhere [11]. The Rotterdam Study (RS) includes three sub-cohorts RS-I, RS-II, and RS-III. Baseline data were collected from 1989 to 1992 in RS-I (*n* = 7983), from 2000 to 2003 in RS-II (*n* = 3011), and from 2006 to 2009 in RS-III (*n* = 3932). Follow-up examinations were conducted periodically, which consisted of a home interview and an extensive set of tests at the research facility. In addition, the data from the medical records of the general practitioners (GPs), nursing homes, and hospitals were collected. The Medical Ethics Committee of the Erasmus Medical Center approved the Rotterdam Study, and written consent was obtained from all participants. The study population for our analysis consisted of all participants with COPD who gave informed consent for follow-up monitoring and had pharmacy, genetic, and covariables data available until 1 January 2011.

### 2.2. COPD and COPD Exacerbations

The diagnosis of COPD was confirmed by pre-bronchodilator obstructive spirometry (forced expiratory volume in 1 s (FEV1)/forced vital capacity (FVC) < 0.7) [12]. In case spirometry was uninterpretable, COPD cases were diagnosed by a physician based on clinical history, physical examination, and spirometry [12]. COPD diagnosed prior to study start was defined as prevalent COPD, and incident COPD was defined as the first diagnosis of COPD during follow-up.

Subjects were followed from cohort entry or the date of COPD diagnosis (incident COPD) until the first COPD exacerbation, death, lost to follow-up, or the end of the study period (i.e., 1 January 2011), whichever came first. A moderate COPD exacerbation was defined as an acute episode of worsening of COPD symptoms requiring a course of systemic corticosteroid and/or antibiotics [13]. If a patient was hospitalized because of COPD exacerbation, it was classified as a severe COPD exacerbation [13]. The first COPD exacerbation was defined as the outcome of interest and the date of outcome was taken as the index date.

### 2.3. Drug Exposure

Medication dispensing data were obtained from the computerized pharmacies in the study district. Records of all filled prescriptions from 1 January 1991 onwards were available and included information on the product name, the Anatomical Therapeutic Chemical Classification (ATC) codes [14], the dispensing date, the prescribed dosing regimen, and the amount dispensed. The studied β_2_-agonists inhalers comprised of (i) short-acting β_2_-agonists (SABA): salbutamol either in monotherapy (R03AC02) or as a fixed-dose combination with ipratropium bromide (R03AL02), terbutaline (R03AC03), fenoterol either in monotherapy (R03AC04) or as a fixed-dose combination with ipratropium bromide (R03AL01), and (ii) long-acting β_2_-agonists (LABA): salmeterol either in monotherapy (R03AC12) or as a fixed-dose combination with fluticasone (R03AK06), formoterol either in monotherapy (R03AC13) or as a fixed-dose combination with budesonide (R03AK07) or with beclometasone (R03AK08). The newer β_2_-agonists inhalers like indacaterol or olodaterol either in monotherapy or as a fixed-dose combination with inhaled corticosteroid (ICS) were not yet available on the Dutch market at the time the study was conducted. To investigate a dose-response relationship, the prescribed daily dose of each β_2_-agonist was expressed in standardized defined daily doses according to the ATC/DDD-stem of the World Health Organization (DDDs) [14]. Patients were considered as “current users” if they used a β_2_-agonist on the index date or when the last use of β_2_-agonists fell within 14 days prior to the index date. If the date of last use of β_2_-agonists was more than 14 days prior to the index date, subjects were considered as “past users”. Patients were considered as “non-users” if they had never used β_2_-agonists prior to the index date during the study period. Data on ICS use, as monotherapy and/or fixed-dose combination with LABA, were extracted from pharmacy records with ATC codes (R03BA, R03AK06, R03AK07, and R03AK08). ICS users were compared to non-users as a reference group.

### 2.4. Genotyping

Subjects in RS were genotyped with Illumina 500 (+duo) and Illumina Human 610-Quad BeadChips. The quality control (QC) procedures were applied. The genotype data were imputed with the 1000-Genomes reference panel (phase 1, V.3) using MACH V.1.0.15/1.0.16. We extracted genotype dosages for two SNPs rs1042713 (16Arg > Gly) and rs1042714 (27Gln > Glu) within the *ADRB2* gene. Imputation quality for both SNPs was high (>0.99).

### 2.5. Functional Annotation of Variants and Expression Quantitative Trait Loci (eQTL) Analysis

We retrieved all proxy SNPs in high linkage disequilibrium (LD) (*r*^2^ threshold > 0.8, limit distance 100 kb, and population panel CEU) with the *ADRB2* variants; rs1042713 and rs1042714. For the functional annotation of the variants, we checked their predicted functions, including effects on gene regulation, protein structure, and splicing by using the HaploRegv4.1 (https://www.broadinstitute.org/mammals/haploreg/haploreg.php) [15]. The correlation of the SNPs and its proxies in high LD with the expression level of the *ADRB2* gene in whole blood was checked using expression quantitative trait loci (eQTL) data from GeneNetwork [16].

### 2.6. Covariables

Covariables consisted of age, sex, smoking, use of ICS, and the daily dose of β_2_-agonists. Data on smoking were obtained from questionnaires and were categorized into “never” or “ever-smokers”. Further details are described in the Supplementary Methods.

### 2.7. Systematic Review

We conducted an extensive electronic literature search of Embase, Medline Ovid, and Cochrane Central using multiple search terms (Supplementary Table S1) to identify all articles investigating the association between the *ADRB2* polymorphisms of interest, namely rs1042713 and/or rs1042714 and the risk of COPD exacerbation in patients treated with inhaled β_2_-agonists. Our literature search was restricted to studies published in English from inception until 30 September 2019. Further details are described in the Supplementary Methods.

### 2.8. Statistical Analysis

Cox proportional hazards models were used to calculate hazard ratios (HRs) and their 95% confidence intervals (CIs) to analyze the association between each polymorphism of the *ADRB2* gene (as well as their haplotypes) and time to first COPD exacerbation. The exposure status to inhaled β_2_-agonists was analyzed as a time-dependent variable [17]. The model estimates the exposure status of the case to inhaled β_2_-agonists on the event date (index date) and the exposure status of all other participants in the cohort on the same date of follow-up [17]. Thus, each stratum consisted of one case and all other cohort participants who were event-free on the index date and still in follow-up [17]. To account for potential confounding by indication, we stratified the study population into three categories, namely current users, past users, and non-users as defined in the methods section. An additive genetic model was assumed for the analysis. For SNPs analyses, we included rs1042713 and rs1042714 separately in the models and adjusted for age, sex, and smoking in the total cohort of COPD patients. In the categories of non-users and past users of β_2_-agonists, we adjusted for age, sex, ICS use, and smoking. The model was further adjusted for the daily dose of β_2_-agonists as a continuous variable in the category of current users.

The Haploview 4.2 [18] was used to estimate haplotypes frequencies and linkage disequilibrium (LD) between two SNPs. The haplo.stats package [19] (version 1.7.7) for R was applied to analyze the association between haplotypes and COPD exacerbations. The statistical methods of the haplo.stats package assume that all subjects are unrelated and linkage phase of the genetic markers is unknown [19]. The haplo.design function [19] was used to calculate haplotype effects for the haplotypes: Arg16/Gln27 and Gly16/Gln27 in reference to the baseline effect of the most frequent haplotype (Gly16/Glu27).

Most studies evaluated the effect of polymorphisms of the *ADRB2* gene among COPD patients with a smoking history. Hence, we investigated the association in ever-smokers. Sensitivity analyses were performed to evaluate the effect of *ADRB2* polymorphisms in the strata of current users of SABA only and LABA only. Because two SNPs (rs1042713 and rs1042714) were investigated, a Bonferroni-corrected P-value lower than 2.5 × 10^−2^ (0.05/2) was considered statistically significant. The data were analyzed using the SPSS statistical software version 24 (IBM SPSS Statistics for Windows; IBM Corp, Armonk, NY, USA) and R package (version 3.3.3) for haplotype analysis using the haplo.stats.

## 3. Results

### 3.1. Characteristics of the Study Population

The study flow of participants is described in the Supplementary Figure S1. Table 1 shows the baseline characteristics of the study population. The mean age (± SD) was 69.6 ± 9.0 years and 57.1% of subjects were male. At the end of follow-up, 80.0% of the study population (*n* = 842) had at least one COPD exacerbation. The minor allele frequencies for rs1042713 (Arg) and rs1042714 (Glu) were 0.35 and 0.47, respectively. Both SNPs were in Hardy-Weinberg equilibrium and they showed an LD with *r*^2^ = 0.47 (D′ = 1). Three haplotypes were determined at positions 16 and 27, and haplotype frequencies were as follows: Gly16/Glu27 (0.48), Arg16/Gln27 (0.35), and Gly16/Gln27 (0.17).

### 3.2. Association of ADRB2 Polymorphisms and COPD Exacerbations

In current β_2_-agonist users, the risk of COPD exacerbation decreased by 30% (HR: 0.70, 95% CI; 0.59–0.84) for each copy of the Arg allele of rs1042713 and by 20% (HR: 0.80, 95% CI; 0.69–0.94) for each copy of the Gln allele of rs1042714 in the adjusted models (Table 2). The rs1042713 and rs1042714 polymorphisms were not associated with the risk of COPD exacerbation in the total cohort of COPD patients (irrespective of β_2_-agonists use) as well as in non-users and past users of inhaled β_2_-agonists (Table 2).

**Figure 1:**
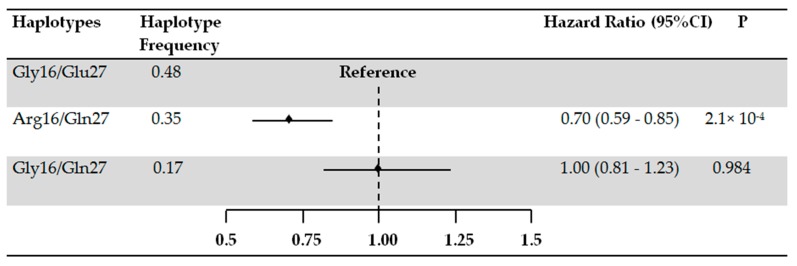
*ADRB2* haplotypes and the risk of COPD exacerbations in current users of β_2_-agonists. The effect of Arg16/Gln27 and Gly16/Gln27 haplotypes compared to the effect of Gly16/Glu27 haplotype. The analyses were adjusted for age, sex, smoking, use of inhaled corticosteroids, and the daily dose of β_2_-agonists.

To explore the combined effect of the two SNPs, we performed haplotype analysis (Figure 1). In the adjusted model, current β_2_-agonist users carrying the Arg16/Gln27 haplotype had a reduced risk of COPD exacerbation (HR: 0.70, 95% CI; 0.59–0.85) compared to the Gly16/Glu27 haplotype. No protective effect of the Gly16/Gln27 haplotype on COPD exacerbation could be observed (Figure 1).

Haploreg v4.1 data showed that rs1042713 and rs1042714 have no non-synonymous proxy variants in strong LD (*r*^2^ > 0.8) (Supplementary Tables S2 and S3). Moreover, the cis-eQTL data form GeneNetwork showed that the Arg allele (A) of rs1042713 and the Gln allele (C) of rs1042714 are associated with reduced levels of the *ADRB2* gene in whole blood [16].

### 3.3. Sensitivity Analyses

**Figure 2:**
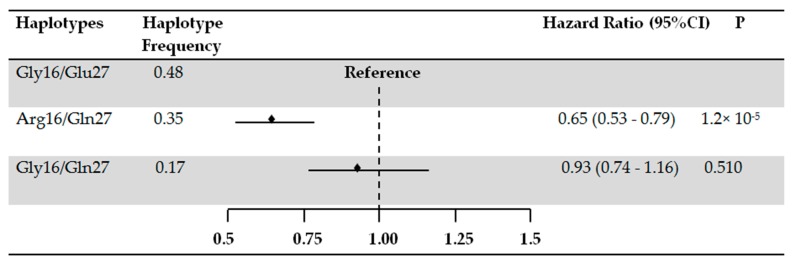
*ADRB2* haplotypes and the risk of COPD exacerbations in current users of β_2_-agonists (smokers only). The effect of Arg16/Gln27 and Gly16/Gln27 haplotypes compared to the effect of Gly16/Glu27 haplotype. The analyses were adjusted for age, sex, use of inhaled corticosteroids, and the daily dose of β_2_-agonists.

We repeated the analysis by excluding never-smokers from our cohort of current users of β_2_-agonists (Table 3 and Figure 2). The results of SNPs and haplotypes analyses remained statistically significant and with similar risk estimates as for the main analyses. When we performed the analysis in strata of current users of SABA only and LABA only, we observed a statistically significantly reduced risk of COPD exacerbations per copy of the Arg allele of rs1042713 among current users of SABA (Table 4). In the LABA only treatment category, we observed a similar trend as in the main analysis; however, the estimates lacked statistical significance (Table 4).

### 3.4. Systematic Review

A flow chart (Supplementary Figure S2) describes study identification, screening, and inclusion. Three clinical trials, as well as four observational studies that investigated the association of interest, met the inclusion criteria. Due to differences in assessments and definitions of the outcome, data could not be pooled (Table 5). Details of the results of the systematic review are provided in the Supplementary Materials.

## 4. Discussion

In this population-based cohort study, we observed that *ADRB2* polymorphisms: rs1042713 and rs1042714 were associated with a reduced risk of COPD exacerbation in current users of inhaled β_2_-agonists. Also, among current users of β_2_-agonist, carriers of the Arg16/Gln27 haplotype had a significantly lower risk of COPD exacerbation compared to those with the Gly16/Glu27 haplotype.

To the best of our knowledge, this is the first population-based study assessing the association between *ADRB2* polymorphisms and COPD exacerbations in patients with COPD treated with inhaled β_2_-agonists. In a substudy of the POET-COPD trial [7] a one year randomized, double-blind, and double-dummy trial found that amongst patients treated with salmeterol, those with the Arg/Arg genotype of rs1042713 had a reduced risk of COPD exacerbations compared to patients with the Arg/Gly and Gly/Gly genotypes which is in line with our findings [7]. However, the findings of other clinical trials [5,8] showed no significant associations between *ADRB2* polymorphisms and the number of COPD exacerbations in LABA users [5,8]. The clinical trials which were included in our systematic review [5,7,8] (Table 5) investigated the effect of *ADRB2* polymorphism and the risk of COPD exacerbations in patients exposed to LABA whereas we assessed the effect of *ADRB2* polymorphisms among inhaled β_2_-agonists users irrespective whether this was a SABA or a LABA. In a sensitivity analysis, we investigated this association in LABA users only and similar findings as for the main analysis were observed, although these results were no longer statistically significant; this, in turn, can be explained by the small sample size in this particular treatment category. A recent observational study, in spirometry-confirmed COPD patients, examined the associations between *ADRB2* polymorphisms (Arg16Gly and Gln27Glu) and risk of severe COPD exacerbations. [20]. The results of the study showed an increased risk of COPD exacerbations in carriers of Arg16 and Gln27 [20]. However, the proportion of COPD patients treated with LABA from the Copenhagen General Population Study was low (9.8%) [20] particularly in comparison to our finding that revealed a protective effect in the category of current users of inhaled β_2_-agonists. So far, a few studies have examined the association between *ADRB2* haplotypes and response to β_2_-agonist [9,21,23]. A study in Egypt [21] of patients with COPD (*n* = 61), assessed the association between *ADRB2* haplotypes and COPD exacerbations. In contrast to our findings, they showed that the Arg16 genotypes and haplotype were associated with frequent COPD exacerbations. However, not all of COPD patients in this study were on regular β_2_-agonist treatment (88% exposed), and the definition used for COPD exacerbations was not provided [21].

To summarize, a number of studies have assessed the effect of *ADRB2* polymorphisms on treatment response to β_2_-agonists with inconsistent results [5,6,7,8,9,20,21,22,23,24,25]. Variation in the results might be related to differences in the study populations, study designs, ethnicity, outcome definitions, treatment classifications, concomitant drugs, as well as power-related issues due to different sample sizes.

The mechanism by which *ADRB2* polymorphisms confer risk for COPD exacerbations in patients treated with inhaled β_2_-agonists is still unknown. Green et al. conducted in-vitro experiments in human airway smooth muscle cells and showed that cells expressing Arg allele at rs1042713 in *ADRB2* underwent less downregulation in response to long-term β_2_-agonist exposure compared to cells expressing Gly allele at this position in *ADRB2* [26]. This is in line with our findings showing a reduced risk of COPD exacerbations in carriers of the Arg allele treated with β_2_-agonist.

In contrast to COPD, previous studies in asthmatic patients suggested that the Arg allele (A) of rs1042713 was associated with an increased risk of asthma exacerbations in children and young adults [27,28]. Indeed, COPD and asthma have been defined as two distinct diseases. COPD is characterized by persistent respiratory symptoms while in asthma, respiratory symptoms vary over time and also in intensity [1,29]. Furthermore, exacerbations are typically triggered by allergens and infections in patients with asthma and COPD, respectively. [1,29] However, it is still unclear how the SNP would be differently associated with exacerbations in patients with COPD compared to asthmatic patients.

The strengths of the Rotterdam Study are the prospective, population-based cohort design with an extended follow-up. Data were prospectively collected through consistent procedures for all subjects, independent of research questions or upcoming diseases, which made it less prone to selection and information bias.

A potential limitation of our study is the fact that spirometry data were only available from 2002 onwards. Therefore, it could result in an underestimation of asymptomatic COPD in the Rotterdam Study before January 2002. In addition, reversibility tests were not performed which might lead to an overestimation of the prevalence of COPD [30,31]. To overcome this limitation, patients with asthma diagnosis were identified and excluded [12]. Furthermore, smoking status was assessed at the time of visiting the center and not at the index date, implying potential misclassification of smoking status; however, smoking status was categorized into ever and never-smokers. Misclassification would only occur if non-smokers start to smoke during follow-up, which is unlikely in COPD patients. Also, we might have overestimated the use of β_2_-agonists as the exposure was based on dispensing data and not on actual intake. We obtained haplotype frequency estimates using the expectation-maximization (E-M) algorithm. Despite some concerns regarding the accuracy of the methods using phase-unknown data, previous studies have confirmed the usefulness of the haplotype approach [32] and the validity of the statistical technique [33] based on phase-unknown data of unrelated individuals. Moreover, as gene expression and eQTL are tissue-specific, in an optimal setting, they should be examined in lung tissue of COPD patients treated with inhaled β_2_-agonists.

In conclusion, we demonstrated that the Arg16/Gln27 haplotype in *ADRB2* was associated with a reduced risk of exacerbation in COPD patients treated with inhaled β_2_-agonists. However, further research is needed to confirm these findings.

## Figures and Tables

**Table 1:** Baseline characteristics of COPD subjects.

Characteristics	COPD Subjects
*n*	1053
Age (years), mean (SD)	69.6 ± 9.0
Sex (Male), no. (%)	601 (57.1)
Ever smoker *, no. (%)	891 (84.6)
Status at the end of follow up, no. (%)	
Individuals with COPD exacerbation	842 (80.0)
Individuals without COPD exacerbation	211 (20.0)
BMI kg/m^2^, median (IQR)	25.9 (4.7)
Heart failure, no. (%)	82 (7.8)
Coronary heart diseases, no. (%)	132 (12.5)
Hypertension *, no. (%)	575 (54.6)
Diabetes mellitus, no. (%)	83 (7.9)
Minor allele (A) frequency (rs1042713)	0.35
rs1042713 genotype, no. (%)	
Arg/Arg (AA)	134 (12.7)
Arg/Gly (AG)	473 (44.9)
Gly/Gly (GG)	446 (42.4)
Minor allele (G) frequency (rs1042714)	0.47
rs1042714 genotype, no. (%)	
Glu/Glu (GG)	232 (22.0)
Glu/Gln (GC)	536 (50.9)
Gln/Gln (CC)	285 (27.1)
Haplotypes frequency	
Gly16/Glu27	0.48
Arg16/Gln27	0.35
Gly16/Gln27	0.17

SD: standard deviation; BMI: body mass index; IQR: Interquartile Range (the difference between 75th and 25th percentiles). * Data were missing on smoking in two subjects and on hypertension in 146 subjects.

**Table 2:** *ADRB2* polymorphisms (per copy of the effect allele) and the risk of COPD exacerbations.

Db SNP No. *	Effect Allele	Events ^1^	Crude Model		Adjusted Model	
			HR (95% CI)	P	HR (95% CI)	P
Total COPD Population (irrespective of inhaled β_2_-agonist use)						
rs1042713	Arg ^2^	*n* = 842	0.93 (0.84–1.02)	NS	0.93 (0.84–1.02)	NS
rs1042714	Gln ^3^	*n* = 842	0.97 (0.88–1.06)	NS	0.97 (0.89–1.07)	NS
Non-users of inhaled β_2_-agonist						
rs1042713	Arg ^2^	*n* = 375	1.02 (0.88–1.18)	NS	0.98 (0.85–1.13)	NS
rs1042714	Gln^3^	*n* = 375	1.05 (0.91–1.21)	NS	1.05 (0.91–1.21)	NS
Past users of inhaled β_2_-agonists						
rs1042713	Arg ^2^	*n* = 154	0.96 (0.76–1.22)	NS	1.03 (0.81–1.31)	NS
rs1042714	Gln ^3^	*n* = 154	0.88 (0.70–1.11)	NS	0.97 (0.76–1.23)	NS
Current users of inhaled β_2_-agonists						
rs1042713	Arg ^2^	*n* = 313	0.70 (0.59–0.82)	3.1 × 10^−5^	0.70 (0.59–0.84)	9.2 × 10^−5^
rs1042714	Gln ^3^	*n* = 313	0.80 (0.69–0.94)	5.9 × 10^−3^	0.80 (0.69–0.94)	7.2 × 10^−3^

* Seattle single nucleotide polymorphisms (SNPs) database number. ^1^ Events, COPD exacerbations; HR, Hazard ratio. ^2^ Arg (A) allele frequency: 0.35. ^3^ Gln (C) allele frequency: 0.53. NS; non-significant. Additive genetic model was used for analyses. In total COPD population; adjusted for age, sex, and smoking. In non and past-users of β_2_-agonist; adjusted for age, sex, smoking, and use of inhaled corticosteroids. In current-users; adjusted for age, sex, smoking, use of inhaled corticosteroids, and the daily dose of β_2_-agonists.

**Table 3:** *ADRB2* polymorphisms (per copy of the effect allele) and the risk of COPD exacerbations in COPD population in current-users of β_2_-agonists (smokers only).

Db SNP No. *	Effect Allele	Events ^1^	Crude Model		Adjusted Model	
			HR (95% CI)	P	HR (95% CI)	P
rs1042713	Arg ^2^	*n* = 277	0.64 (0.53–0.77)	1.9 × 10^−6^	0.66 (0.55–0.80)	1.2 × 10^−5^
rs1042714	Gln ^3^	*n* = 277	0.73 (0.62–0.86)	2.1 × 10^−4^	0.74 (0.63–0.87)	3.8 × 10^−4^

* Seattle single nucleotide polymorphism (SNP) database number.^1^ Events, COPD exacerbations; HR, hazard ratio. ^2^ Arg (A) allele frequency: 0.35. ^3^ Gln (C) allele frequency: 0.53. Additive genetic model was used for analyses. The analyses were adjusted for age, sex, use of inhaled corticosteroids, and the daily dose of β_2_-agonists.

**Table 4:** *ADRB2* polymorphisms (per copy of the effect allele) and the risk of COPD exacerbations in current-users of SABA only or LABA only.

Db SNP No. *	Effect Allele	Events ^1^	Crude Models		Adjusted Models	
			HR (95% CI)	P	HR (95% CI)	P
SABA only						
rs1042713	Arg ^2^	*n* = 205	0.73 (0.59–0.90)	2.9 × 10^−3^	0.72 (0.58–0.90)	3.0 × 10^−3^
rs1042714	Gln ^3^	*n* = 205	0.81 (0.67–0.99)	3.6 × 10^−2^	0.80 (0.66–0.98)	3.0 × 10^−2^
LABA only						
rs1042713	Arg ^2^	*n* = 85	0.73 (0.53–1.03)	7.1 × 10^−2^	0.70 (0.48–0.98)	4.0 × 10^−2^
rs1042714	Gln ^3^	*n* = 85	0.91 (0.67–1.22)	0.525	0.92 (0.67–1.27)	0.631

* Seattle single nucleotide polymorphism (SNP) database number. ^1^ Events, COPD exacerbations; SABA, short-acting β_2_-agonists; LABA, long-acting β_2_-agonists; HR, Hazard ratio. ^2^ Arg (A) allele frequency: 0.35. ^3^ Gln (C) allele frequency: 0.53. Additive genetic model was used for analyses. Adjusted model: adjusted for age, sex, use of inhaled corticosteroids, the daily dose of β_2_-agonists and smoking.

**Table 5:** Overview of the studies included in the review.

Study (Year)	Design	Study Population	Country	Treatment	Outcome	Definition of COPD Exacerbation	SNP(s)	Estimate/Association
All participants were on β_2_-agonists treatment								
Rabe et al. (2014) [7]	Randomized controlled trial	2561 COPD patients with a history of smoking	Multi-center in 25 countries	Salmeterol plus inhaled corticosteroids	Time to first COPD exacerbation; Kaplan-Meier curves were produced and the log-rank test was used for comparison.	Need of antibiotics or systemic glucocorticoids or admission to hospital	rs1042713 rs1042714	rs1042713: Arg16Arg genotype was associated with reduced risk of exacerbation compared to Gly16Gly and Arg16Gly genotypes rs1042714: no association
Bleeker et al. (2012) [8]	Two randomized controlled trials	Study 1, 1456 Study2, 1383 COPD patients with a history of smoking	Multi-center (US, Europe and Mexico)	Formoterol only or in combination with budesonide	Number of COPD exacerbations per patient-treatment year	Need of oral corticosteroid treatment or hospitalization	rs1042713	No association between rs1042713 genotypes and number of COPD exacerbations per patient-treatment year
Yelensky et al. (2012) [5]	Retrospective analysis of phase III clinical trials	565 COPD patients with a history of smoking	USA	Patients treated with Indacaterol for 26 weeks	Number of COPD exacerbations during the 26-week of treatment; using Poisson regression	Need of systemic glucocorticoid therapy, antibiotics, oxygen treatment and/or hospitalization or emergency room visit.	rs1042711 rs1042713 rs1042714 rs1800888	No association between the SNPs and number of COPD exacerbations.
Not all participants were on β_2_-agonists treatment								
Ingebrigtsen et al. (2019) [20]	Prospective cohort	5219 COPD patients and 85.3% of them had a history of smoking (Copenhagen General Population Study)	Denmark	9.8 % of COPD patients were on LABA treatment	Time to first exacerbation; by using univariable competing risks regression analyses	As acute admissions with a discharge diagnosis of COPD	rs1042713 rs1042714	The Arg allele at rs1042713 and the Gln allele at rs1042714 were associated with an increased risk of COPD exacerbations
Hussein et al. (2017) [21]	Case-control study	61 COPD patients with a history of smoking, (recruited from three hospitals)	Egypt	88% of patients were on β_2_-agonists treatment	Number of exacerbations	No definition for COPD exacerbation	rs1042713 rs1042714	rs1042713: Arg16 genotypes and haplotypes were associated with more frequent exacerbations.
Emeryk-Mksymiuk et al. (2017) [6]	Retrospective study	92 COPD patients with a history of smoking, (recruited from outpatient clinic)	Poland	83% of patients were on β_2_-agonists treatment	Self-reported exacerbations	Need of antibiotic therapy, systemic glucocorticoid therapy or hospitalization	rs1042713 rs1042714	rs1042713: patients with Arg/Arg genotype required more frequent treatment with antibiotics, as well as systemic corticosteroid therapy. rs1042714: no association
Vacca et al. (2009) [22]	Case-control study	190 COPD patients with a history of smoking (recruited from two centers)	Germany	No information on β_2_-agonist treatment	≥3 exacerbations within the last 3 year vs no exacerbation within the last 2 years	Need of hospitalization	rs1042713 rs1042714	rs1042713: no association reported rs1042714: no association reported

## Supplementary Materials

The following are available online at https://www.mdpi.com/2077-0383/8/11/1835/s1.

## References

[1] (2019). Global Strategy for Diagnosis, Management, and Prevention of COPD. https://goldcopd.org/gold-reports/.

[2] Johnson M. (2006). Molecular mechanisms of beta(2)-adrenergic receptor function, response, and regulation. J. Allergy Clin. Immunol..

[3] McGraw D.W., Liggett S.B. (2005). Molecular mechanisms of beta2-adrenergic receptor function and regulation. Proc. Am. Thorac. Soc..

[4] Ortega V.E., Hawkins G.A., Moore W.C., Hastie A.T., Ampleford E.J., Busse W.W., Castro M., Chardon D., Erzurum S.C., Israel E. (2014). Effect of rare variants in ADRB2 on risk of severe exacerbations and symptom control during longacting beta agonist treatment in a multiethnic asthma population: A genetic study. Lancet Respir. Med..

[5] Yelensky R., Li Y., Lewitzky S., Leroy E., Hurwitz C., Rodman D., Trifilieff A., Paulding C.A. (2012). A pharmacogenetic study of ADRB2 polymorphisms and indacaterol response in COPD patients. Pharm. J..

[6] Emeryk-Maksymiuk J., Emeryk A., Krawczyk P., Wojas-Krawczyk K., Milanowski J. (2017). Beta-2-adrenoreceptor polymorphism at position 16 determines the clinical severity of chronic obstructive pulmonary disease. Pulm. Pharmacol. Ther..

[7] Rabe K.F., Fabbri L.M., Israel E., Kogler H., Riemann K., Schmidt H., Glaab T., Vogelmeier C.F. (2014). Effect of ADRB2 polymorphisms on the efficacy of salmeterol and tiotropium in preventing COPD exacerbations: A prespecified substudy of the POET-COPD trial. Lancet Respir. Med..

[8] Bleecker E.R., Meyers D.A., Bailey W.C., Sims A.M., Bujac S.R., Goldman M., Martin U.J. (2012). ADRB2 polymorphisms and budesonide/formoterol responses in COPD. Chest.

[9] Hizawa N., Makita H., Nasuhara Y., Betsuyaku T., Itoh Y., Nagai K., Hasegawa M., Nishimura M. (2007). Beta2-adrenergic receptor genetic polymorphisms and short-term bronchodilator responses in patients with COPD. Chest.

[10] Kim W.J., Oh Y.M., Sung J., Kim T.H., Huh J.W., Jung H., Lee J.H., Kim E.K., Lee S.M., Lee S. (2008). Lung function response to 12-week treatment with combined inhalation of long-acting beta2 agonist and glucocorticoid according to ADRB2 polymorphism in patients with chronic obstructive pulmonary disease. Lung.

[11] Hofman A., Brusselle G.G., Darwish Murad S., van Duijn C.M., Franco O.H., Goedegebure A., Ikram M.A., Klaver C.C., Nijsten T.E., Peeters R.P. (2015). Epidemiology and impact of chronic bronchitis in chronic obstructive pulmonary disease. Eur. J. Epidemiol..

[12] Terzikhan N., Verhamme K.M., Hofman A., Stricker B.H., Brusselle G.G., Lahousse L. (2016). Prevalence and incidence of COPD in smokers and non-smokers: The Rotterdam Study. Eur. J. Epidemiol..

[13] Lahousse L., Seys L.J.M., Joos G.F., Franco O.H., Stricker B.H., Brusselle G.G. (2017). Epidemiology and impact of chronic bronchitis in chronic obstructive pulmonary disease. Eur. Respir. J..

[14] WHO (2018). WHO Collaborating Centre for Drug Statistics Methodology. https://www.whocc.no/atc_ddd_index/.

[15] (2015). HaploReg v4.1, Broad Institute. https://broadinstitute.org/mammals/haploreg/haploreg.php/.

[16] Westra H.J., Peters M.J., Esko T., Yaghootkar H., Schurmann C., Kettunen J., Christiansen M.W., Fairfax B.P., Schramm K., Powell J.E. (2013). Systematic identification of trans eQTLs as putative drivers of known disease associations. Nat. Genet..

[17] Stricker B.H., Stijnen T. (2010). Analysis of individual drug use as a time-varying determinant of exposure in prospective population-based cohort studies. Eur. J. Epidemiol..

[18] Barrett J.C., Fry B., Maller J., Daly M.J. (2005). Haploview: Analysis and visualization of LD and haplotype maps. Bioinformatics.

[19] Sinnwell J.P., Schaid D.J. (2016). Statistical Analysis of Haplotypes with Traits and Covariates When Linkage Phase Is Ambiguous. R Package Version 1.7.7. https://CRAN.R-project.org/package=haplo.stats.

[20] Ingebrigtsen T.S., Vestbo J., Rode L., Marott J.L., Lange P., Nordestgaard B.G. (2019). β2-Adrenergic genotypes and risk of severe exacerbations in COPD: A prospective cohort study. Thorax.

[21] Hussein M.H., Sobhy K.E., Sabry I.M., El Serafi A.T., Toraih E.A. (2017). Beta2-adrenergic receptor gene haplotypes and bronchodilator response in Egyptian patients with chronic obstructive pulmonary disease. Adv. Med Sci..

[22] Vacca G., Schwabe K., Duck R., Hlawa H.P., Westphal A., Pabst S., Grohe C., Gillissen A. (2009). Polymorphisms of the beta2 adrenoreceptor gene in chronic obstructive pulmonary disease. Ther. Adv. Respir. Dis..

[23] Mokry M., Joppa P., Slaba E., Zidzik J., Habalova V., Kluchova Z., Micietova L., Rozborilova E., Salagovic J., Tkacova R. (2008). Beta2-adrenergic receptor haplotype and bronchodilator response to salbutamol in patients with acute exacerbations of COPD. Med. Sci. Monit..

[24] Konno S., Makita H., Hasegawa M., Nasuhara Y., Nagai K., Betsuyaku T., Hizawa N., Nishimura M. (2011). Beta2-adrenergic receptor polymorphisms as a determinant of preferential bronchodilator responses to beta2-agonist and anticholinergic agents in Japanese patients with chronic obstructive pulmonary disease. Pharm. Genom..

[25] Mochizuki H., Nanjo Y., Kawate E., Yamazaki M., Tsuda Y., Takahashi H. (2012). beta2-adrenergic receptor haplotype may be associated with susceptibility to desensitization to long-acting beta2-agonists in COPD patients. Lung.

[26] Green S.A., Turki J., Bejarano P., Hall I.P., Liggett S.B. (1995). Influence of beta 2-adrenergic receptor genotypes on signal transduction in human airway smooth muscle cells. Am. J. Respir. Cell Mol. Biol..

[27] Turner S., Francis B., Vijverberg S., Pino-Yanes M., Maitland-van der Zee A.H., Basu K., Bignell L., Mukhopadhyay S., Tavendale R., Palmer C. (2016). Childhood asthma exacerbations and the Arg16 beta2-receptor polymorphism: A meta-analysis stratified by treatment. J. Allergy Clin. Immunol..

[28] Basu K., Palmer C.N., Tavendale R., Lipworth B.J., Mukhopadhyay S. (2009). Adrenergic beta(2)-receptor genotype predisposes to exacerbations in steroid-treated asthmatic patients taking frequent albuterol or salmeterol. J. Allergy Clin. Immunol..

[29] Global Strategy for Asthma Management and Prevention, 2019 GINA Report. https://ginasthma.org/gina-reports/.

[30] Tilert T., Dillon C., Paulose-Ram R., Hnizdo E., Doney B. (2013). Estimating the U.S. prevalence of chronic obstructive pulmonary disease using pre- and post-bronchodilator spirometry: The National Health and Nutrition Examination Survey (NHANES) 2007–2010. Respir. Res..

[31] Johannessen A., Omenaas E.R., Bakke P.S., Gulsvik A. (2005). Implications of reversibility testing on prevalence and risk factors for chronic obstructive pulmonary disease: A community study. Thorax.

[32] Tishkoff S.A., Pakstis A.J., Ruano G., Kidd K.K. (2000). The accuracy of statistical methods for estimation of haplotype frequencies: An example from the CD4 locus. Am. J. Hum. Genet..

[33] Zaykin D.V., Westfall P.H., Young S.S., Karnoub M.A., Wagner M.J., Ehm M.G. (2002). Testing association of statistically inferred haplotypes with discrete and continuous traits in samples of unrelated individuals. Hum. Hered..

