# Supplementary material for: β2-Adrenergic Receptor (ADRB2) Gene Polymorphisms and Risk of COPD Exacerbations: The Rotterdam Study
Source: J Clin Med. 2019 Nov 1;8(11):1835. doi: 10.3390/jcm8111835 (PMC6912270; doi:10.3390/jcm8111835)
Supplement: Supplementary file 1 [file jcm-08-01835-s001.pdf]

β_2-Adrenergic Receptor (*ADRB2*) Gene Polymorphisms and Risk of COPD Exacerbations: the Rotterdam Study

(Online Supplementary Material)

Leila Karimi[1], Lies Lahousse[2,3], Mohsen Ghanbari[3,4], Natalie Terzikhan[3,5] , André G. Uitterlinden[6], Johan van der Lei[1], Guy G. Brusselle[3,5,7], Bruno H. Stricker[3,6], Katia M. C. Verhamme[1,2]

[1] Department of Medical Informatics, Erasmus University Medical Centre, Rotterdam, the Netherlands

[2] Department of Bioanalysis, Ghent University, Ghent, Belgium.

[3]Department of Epidemiology, Erasmus University Medical Centre, Rotterdam, the Netherlands

[4] Department of Genetics, School of Medicine, Mashhad University of Medical Science, Mashhad, Iran

[5] Department of Respiratory Medicine, Ghent University Hospital, Ghent, Belgium.

[6] Department of Internal Medicine, Erasmus University Medical Centre, Rotterdam, the Netherlands

[7] Department of Respiratory Medicine, Erasmus University Medical Centre, Rotterdam, the Netherlands

First author: Leila Karimi

Correspondence:

Prof. dr. B.H. Stricker MMed PhD

Erasmus MC

Department of Epidemiology

Postbus 2040

3000 CA ROTTERDAM

b.stricker@erasmusmc.nl

Contents

Supplemental methods 3

Baseline characteristics 3

Systematic review 3

Review criteria and data extraction 4

Supplemental results 4

Supplementary tables and figures 7

Table S1: Search strategy per library 7

Table S2: Functional annotation of rs1042713 using the HaploRegv4.1 8

Table S3: Functional annotation of rs1042714 using the HaploRegv4.1 9

Figure 1: Flowchart of participants 11

Figure S2: A flow chart describing the steps for including studies in the review 12

Supplemental references 13

Supplemental methods

Baseline characteristics

BMI was calculated as weight divided by height squared (kg/m^2). Diabetes mellitus was defined as a fasting serum glucose concentration of ≥7.0 mmol/L or a non-fasting serum glucose concentration of ≥11.1 mmol/L or the use of blood glucose-lowering medications [1]. Hypertension was defined as a resting blood pressure above 140/90 mmHg or the use of blood pressure-lowering medication. The diagnosis of heart failure was based on follow-up using the medical records of the participants [2]. Coronary Heart Diseases (CHD) was defined as a compound outcome including fatal or nonfatal myocardial infarction or CHD mortality [2].

Systematic review

We conducted an extensive electronic literature search of Embase, Medline Ovid, and Cochrane Central using multiple search terms (Supplementary Table S1) to identify all articles investigating *ADRB2* polymorphisms; rs1042713 and/or rs1042714 and/or their haplotypes and COPD exacerbations in patients exposed to β_2-agonists. Our literature search was restricted to studies published in English from inception until 30 September 2019. Additional potentially relevant articles were searched through article reference lists.

Review criteria and data extraction

We considered all original articles, excluding conference abstracts, editorials, short surveys, and animal studies. We did not set any limits on study design, sample size, location, or follow-up. Studies were included if they met the following three criteria;

(1) COPD patients exposed to inhaled short-acting β_2-agonists (SABA) and/or long-acting β_2-agonists (LABA) were eligible to be included in the review.

(2) The exposure variable of interest was *ADRB2* polymorphisms; rs1042713 and/or rs1042714 and/or their haplotypes.

(3) The outcome of interest was COPD exacerbations. COPD exacerbation was defined as acute episodes of worsening symptoms requiring a course of systemic corticosteroid and/or antibiotics and/or hospitalization and/or emergency room visit .

The first author (LK) screened all studies from their titles and abstracts and excluded those that were not relevant. The full texts of potential papers were assessed independently by two authors (LK and KV). In case of heterogeneity across studies, the results of each study were reported individually.

Supplemental results

The literature search yielded 369 hits, of which 270 unique articles remained after excluding duplicates. Of these 270 articles, the title and abstract were reviewed and 236 articles were excluded (conference abstracts (26), editorials (10), experimental studies (5), short surveys (5) and as they were unrelated to the association between *ADRB2* polymorphisms and treatment response to inhaled β_2-agonist in patient with COPD (190). We reviewed 34 full-text articles and 27 of these were excluded for the following reasons; review article (13), letter (1), focus on other SNPs in *ADRB2* (3), focus on different outcomes (10). In total, three

clinical trials and four observational studies were withheld, but in the latter, not all of the included patients were on treatment with inhaled β_2-agonist (Figure S2).

Briefly, the three clinical trials that met inclusion criteria [3-5] were published between 2012 and 2014. The sample size ranged from 565 to 2,561. Two studies were multicentre, and another one was from the United States. One assessed the association between the SNPs and time to first COPD exacerbation using Kaplan-Meier curves and the log-rank test. [4] Rabe et al. found that patients with the Arg16Arg genotype and using salmeterol and inhaled corticosteroids (ICS) had a significantly lower risk of COPD exacerbations compared with Gly16Gly (p=0·0018) and Arg16Gly (p=0·0130) genotypes [4]. They found no significant differences in exacerbation risk between the genotypes of rs1042714.[4] Two other studies [3,5] assessed the association of the SNP(s) with the number of COPD exacerbations. One of them used Poisson regression to asses this association and while the other study described the distribution of the number of COPD exacerbation across the genotype categories of rs104213. They found no significant association between the SNPs and COPD exacerbations in COPD patients using LABA [3,5].

In our search, four observational studies [6-9] also evaluated the association of the SNP(s) with the number of COPD exacerbations. They were published between 2009 and 2019 and included patients from hospitals, medical centres, outpatient clinics, and the general population. Their sample size ranged from 61 to 5,219. However, not all of the included patients in these four observational studies were on treatment with inhaled β_2-agonist. The results of a recent observational study showed an increased risk of COPD exacerbations in carriers of Arg16 and Gln27 [9]. However, the proportion of COPD patients treated with LABA from the Copenhagen General Population Study was low (9.8 %) [9]. Due to differences in assessments and definitions of the outcome, this precluded a meta-analysis

with pooling of results. Therefore, we reported the findings separately for each study in Table 5 in the main text.

Supplementary tables and figures

Table S1: Search strategy per library

**Embase.com**
('adrb2 gene'/de OR 'adrb2 protein human'/de OR (adrb2 OR adrb-2):ab,ti OR (('beta 2 adrenergic receptor'/de OR 'beta adrenergic receptor'/de OR (((beta OR β OR beta2 OR β2) NEAR/3 adrenerg* NEAR/3 receptor*) OR ((beta OR β OR beta2 OR β2) NEAR/3 (adrenorecept* OR adrenocept* OR agonist*))):ab,ti) AND ('genetics'/exp OR 'genetic parameters'/exp OR 'genetic polymorphism'/exp OR genotype/exp OR 'genetic marker'/exp OR 'genetic association'/de OR 'genome-wide association study'/de OR (haplotype* OR polymorph* OR genetic* OR pharmacogenetic* OR snp OR genom* OR gwas):ab,ti))) AND ('chronic obstructive lung disease'/de OR (copd OR (chronic* NEAR/3 obstruct* NEAR/3 (lung OR pulmonar*))):ab,ti) AND [english]/lim
**Medline Ovid**
(ADRB2 protein, human.nm. OR (adrb2 OR adrb-2).ab,ti. OR ((Receptors, Adrenergic, beta-2/ OR Receptors, Adrenergic, beta/ OR (((beta OR beta2) ADJ3 adrenerg* ADJ3 receptor*) OR ((beta OR beta2) ADJ3 (adrenorecept* OR adrenocept* OR agonist*))).ab,ti.) AND (exp Genetics/ OR Genetics.fs. OR exp Genetic Phenomena/ OR exp Genetic Association Studies/ OR (haplotype* OR polymorph* OR genetic* OR pharmacogenetic* OR snp OR genom* OR gwas).ab,ti.))) AND (Pulmonary Disease, Chronic Obstructive/ OR (copd OR (chronic* ADJ3 obstruct* ADJ3 (lung OR pulmonar*))).ab,ti.) AND english.la.
**Cochrane CENTRAL**
((adrb2 OR adrb-2):ab,ti OR (((((beta OR β OR beta2 OR β2) NEAR/3 adrenerg* NEAR/3 receptor*) OR ((beta OR β OR beta2 OR β2) NEAR/3 (adrenorecept* OR adrenocept* OR agonist*))):ab,ti) AND ((haplotype* OR polymorph* OR genetic* OR pharmacogenetic* OR snp OR genom* OR gwas):ab,ti))) AND ((copd OR (chronic* NEAR/3 obstruct* NEAR/3 (lung OR pulmonar*))):ab,ti)

Table S2: Functional annotation of rs1042713 using the HaploRegv4.1

Chr	pos (hg38)	LD (r^2)	LD (D')	variant	Ref	Alt	EUR freq	Enhancer histone marks	DNAse	Motifs changed	Selected eQTL hits	GENCODE genes
**5**	148819704	0.9	0.95	rs35283004	A	G	0.38	BLD, MUS		GR,Maf	2 hits	6.9kb 5' of *ADRB2*
**5**	148820281	0.81	0.92	rs71582318	T	C	0.37	BLD, SKIN		Pou1f1,TATA		6.3kb 5' of *ADRB2*
**5**	148821442	0.94	0.97	rs12189018	T	C	0.38	BLD		RXRA	2 hits	5.2kb 5' of *ADRB2*
**5**	148822166	0.94	0.97	rs35019280	AG	A	0.38	BLD		CIZ,GATA,HNF1	2 hits	4.4kb 5' of *ADRB2*
**5**	148822926	0.93	0.97	rs33910799	AG	A	0.38	BLD	BD	CEBPB,DMRT2	1 hit	3.7kb 5' of *ADRB2*
**5**	148825014	0.97	0.99	rs17778257	A	T	0.38	9 tissues	SKIN	5 altered motifs	4 hits	1.6kb 5' of *ADRB2*
**5**	148826178	0.96	0.98	rs12654778	G	A	0.38		38 tissues	Foxp3,p53	4 hits	414bp 5' of *ADRB2*
**5**	148826877	1	1	rs1042713	G	A	0.38		28 tissues	4 altered motifs	3 hits	*ADRB2*

Pos, position; **LD,** Linkage disequilibrium; **Ref,** reference; **Alt,** alternative; **EUR freq**, European frequency; **eQTL**, expression quantitative trait loci.

Table S3: Functional annotation of rs1042714 using the HaploRegv4.1

chr	pos (hg38)	LD (r^2)	LD (D')	variant	Ref	Alt	EUR freq	Enhancer histone marks	DNAse	Motifs changed	Selected eQTL hits	GENCODE genes
**5**	148819436	0.88	0.94	rs4705059	C	T	0.59	BLD, HRT, MUS	HRT	5 altered motifs		7.2kb 5' of *ADRB2*
**5**	148819441	0.88	0.94	rs4705060	G	A	0.59	BLD, MUS		4 altered motifs		7.2kb 5' of *ADRB2*
**5**	148819679	0.9	0.96	rs10078004	G	A	0.60			Mrg,NRSF		6.9kb 5' of *ADRB2*
**5**	148819882	0.9	0.96	rs67339154	A	G	0.60	BLD		Brachyury,TBX5		6.7kb 5' of *ADRB2*
**5**	148820448	0.94	0.97	rs56330463	T	C	0.59	BLD, SKIN		PPAR		6.1kb 5' of *ADRB2*
**5**	148820990	0.94	0.98	rs2082382	G	A	0.60	BLD	38 tissues	Foxo,Rad21	2 hits	5.6kb 5' of *ADRB2*
**5**	148821037	0.97	0.99	rs2082395	A	G	0.59	BLD	25 tissues	5 altered motifs	2 hits	5.6kb 5' of *ADRB2*
**5**	148821395	0.95	0.99	rs9325120	C	A	0.58	BLD		4 altered motifs		5.2kb 5' of *ADRB2*
**5**	148821692	0.97	0.99	rs11168066	C	A	0.59	BLD		Dmbx1,Otx2	2 hits	4.9kb 5' of *ADRB2*
**5**	148821753	0.96	0.99	rs11959615	T	A	0.59	BLD			2 hits	4.8kb 5' of *ADRB2*
**5**	148821910	0.97	0.99	rs35875547	AT	A	0.59	BLD, BRN		10 altered motifs		4.7kb 5' of *ADRB2*
**5**	148821922	0.97	0.99	rs11958940	A	T	0.59	BLD, BRN		NRSF,Zbtb3		4.7kb 5' of *ADRB2*
**5**	148822006	0.97	0.99	rs34064454	A	G	0.59	BLD, BRN		AIRE,Pax-4		4.6kb 5' of *ADRB2*
**5**	148823105	0.97	0.99	rs11746634	C	G	0.59	ESC, BLD		LUN-1,RORalpha1		3.5kb 5' of *ADRB2*
**5**	148823238	0.97	0.99	rs11168067	A	G	0.59	BLD		NRSF,Pitx2,SETDB1		3.4kb 5' of *ADRB2*
**5**	148823373	0.95	0.99	rs9325122	C	T	0.60	BLD		HDAC2,Pou2f2,Pou3f3		3.2kb 5' of *ADRB2*
**5**	148824199	0.97	0.99	rs1432622	T	C	0.59	BLD		7 altered motifs	2 hits	2.4kb 5' of *ADRB2*

Table S3. Functional annotation of rs1042714 using the HaploRegv4.1 (cont'd)

chr	pos (hg38)	LD (r^2)	LD (D')	variant	Ref	Alt	EUR freq	Enhancer histone marks	DNAse	Motifs changed	Selected eQTL hits	GENCODE genes
**5**	148824445	0.97	0.99	rs1432623	C	T	0.59	BLD, SKIN		Nkx2		2.1kb 5' of *ADRB2*
**5**	148824558	0.97	0.99	rs11168068	C	T	0.59	BLD, SKIN		8 altered motifs		2kb 5' of *ADRB2*
**5**	148825489	0.97	0.99	rs2400707	A	G	0.59	12 tissues	SKIN,SKIN	HLF	2 hits	1.1kb 5' of *ADRB2*
**5**	148825809	0.97	0.99	rs2053044	A	G	0.59	5 tissues	35 tissues	8 altered motifs		783bp 5' of *ADRB2*
**5**	148826364	0.99	0.99	rs11168070	G	C	0.59		51 tissues	GR		228bp 5' of *ADRB2*
**5**	148826465	0.99	1	rs11959427	C	T	0.59	BRN	52 tissues	11 altered motifs		127bp 5' of *ADRB2*
**5**	148826785	0.98	1	rs1042711	C	T	0.59		35 tissues	6 altered motifs		5'-UTR of *ADRB2*
**5**	148826812	0.98	1	rs1801704	C	T	0.59	BRN	37 tissues	E2A,Sin3Ak-20,ZEB1		5'-UTR of *ADRB2*
**5**	148826910	1	1	rs1042714	G	C	0.59		21 tissues	GATA,PU.1		*ADRB2*

Pos, position; **LD,** Linkage disequilibrium; **Ref,** reference; **Alt,** alternative; **EUR freq**, European frequency; **eQTL**, expression quantitative trait loci

Figure 1: Flowchart of participants

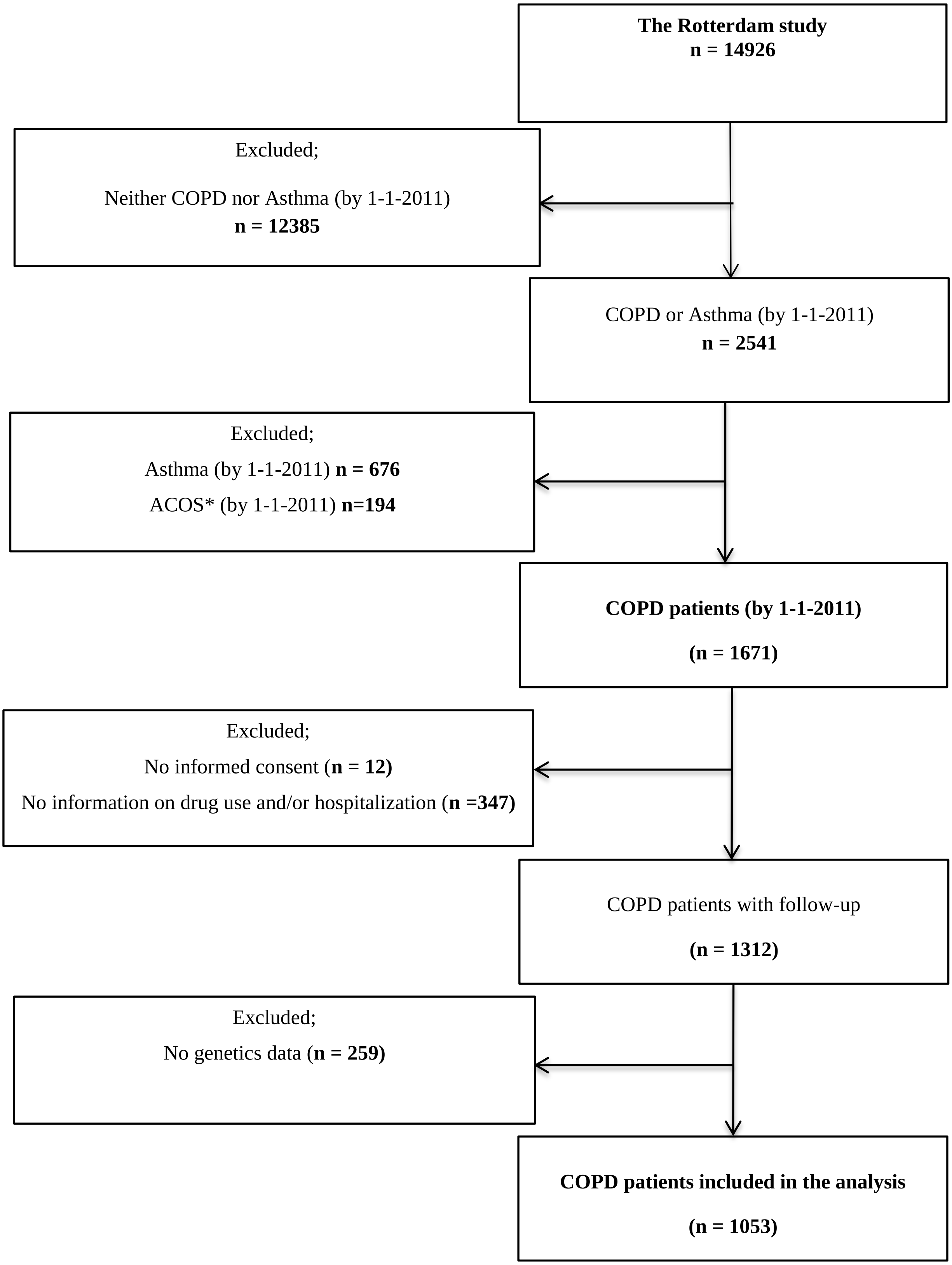

* Asthma and COPD overlap syndrome

Figure S2: A flow chart describing the steps for including studies in the review

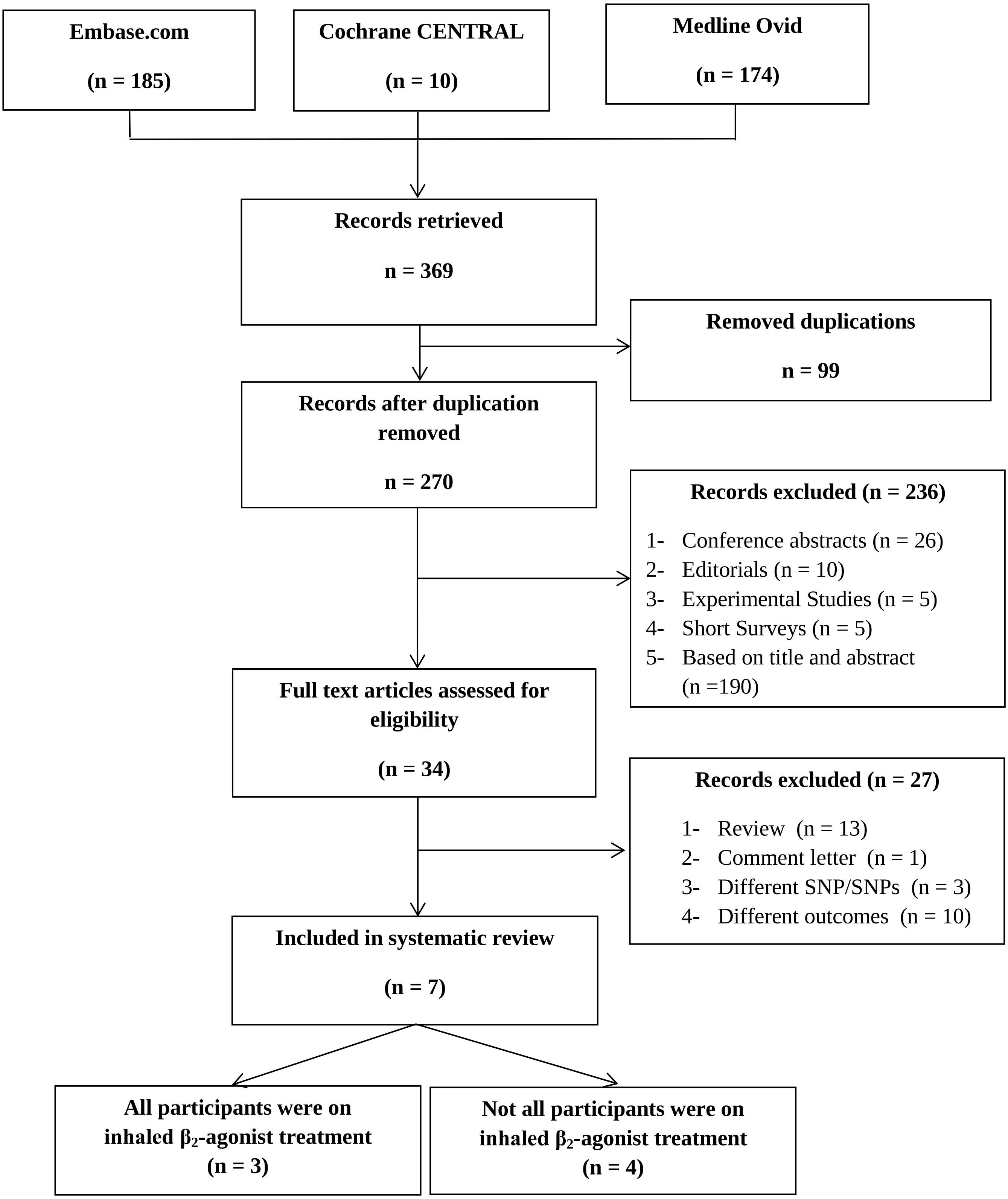

Supplemental references

1. Ligthart S, van Herpt TT, Leening MJ, Kavousi M, Hofman A, Stricker BH, van Hoek M, Sijbrands EJ, Franco OH, Dehghan A. Lifetime risk of developing impaired glucose metabolism and eventual progression from prediabetes to type 2 diabetes: a prospective cohort study. The lancet. Diabetes & endocrinology 2016; 4: 44-51.
2. Leening MJ, Kavousi M, Heeringa J, van Rooij FJ, Verkroost-van Heemst J, Deckers JW, Mattace-Raso FU, Ziere G, Hofman A, Stricker BH, Witteman JC. Methods of data collection and definitions of cardiac outcomes in the Rotterdam Study. European journal of epidemiology 2012; 27: 173-185.
3. Bleecker ER, Meyers DA, Bailey WC, Sims AM, Bujac SR, Goldman M, Martin UJ. ADRB2 polymorphisms and budesonide/formoterol responses in COPD. Chest 2012; 142: 320-328.
4. Rabe KF, Fabbri LM, Israel E, Kogler H, Riemann K, Schmidt H, Glaab T, Vogelmeier CF. Effect of ADRB2 polymorphisms on the efficacy of salmeterol and tiotropium in preventing COPD exacerbations: a prespecified substudy of the POET-COPD trial. The Lancet. Respiratory medicine 2014; 2: 44-53.
5. Yelensky R, Li Y, Lewitzky S, Leroy E, Hurwitz C, Rodman D, Trifilieff A, Paulding CA. A pharmacogenetic study of ADRB2 polymorphisms and indacaterol response in COPD patients. The pharmacogenomics journal 2012; 12: 484-488.
6. Emeryk-Maksymiuk J, Emeryk A, Krawczyk P, Wojas-Krawczyk K, Milanowski J. Beta-2-adrenoreceptor polymorphism at position 16 determines the clinical severity of chronic obstructive pulmonary disease. Pulmonary pharmacology & therapeutics 2017; 43: 1-5.

7. Hussein MH, Sobhy KE, Sabry IM, El Serafi AT, Toraih EA. Beta2-adrenergic receptor gene haplotypes and bronchodilator response in Egyptian patients with chronic obstructive pulmonary disease. Advances in medical sciences 2017; 62: 193-201.

8. Vacca G, Schwabe K, Duck R, Hlawa HP, Westphal A, Pabst S, Grohe C, Gillissen A. Polymorphisms of the beta2 adrenoreceptor gene in chronic obstructive pulmonary disease. Therapeutic advances in respiratory disease 2009; 3: 3-10.

9. Ingebrigtsen TS, Vestbo J, Rode L, Marott JL, Lange P, Nordestgaard BG. β2-Adrenergic genotypes and risk of severe exacerbations in COPD: A prospective cohort study. Thorax 2019.
